# Effect of reserve protection level and governance on tree cover loss and gain

**DOI:** 10.1111/cobi.14449

**Published:** 2025-01-24

**Authors:** Natasha Stoudmann, Jason Byrne, Vanessa Adams

**Affiliations:** ^1^ School of Geography, Planning, and Spatial Sciences University of Tasmania Hobart Tasmania Australia

**Keywords:** conservation effectiveness, impact evaluation, protected areas, statistical matching, terrestrial reserves, tree cover dynamics, áreas protegidas, cotejo estadístico, dinámicas de cobertura forestal, efectividad de la conservación, evaluación de impacto, reservas terrestres

## Abstract

Terrestrial protected areas are essential for biodiversity conservation, yet it is not fully understood when and how different types of protected areas are most effective in achieving specific conservation objectives. We assessed the impact of reserves on tree cover loss and gain through a case study in Tasmania, Australia. We considered varying protection levels (strict, where human activities are restricted, and multiple use) and governance types (public and private). We used a counterfactual matching approach to compare tree cover loss and gain between reserves and matched unprotected areas from 2004 to 2021. We accounted for forest policy changes, environmental covariates, and human pressures to reduce placement bias. We also characterized reserves by size, governance, management, and vegetation and compared covariates inside and outside reserves to define baseline conditions. Reserves established from 2004 to 2016 were overall 75.4% less likely to have lost tree cover and 16.0% more likely to have had tree cover gain compared with controls. Patterns of loss and gain varied by protection level and governance type. Multiple‐use reserves were as effective as reserves in which human activities were more restricted. Privately managed reserves contributed to tree cover growth, and public reserves helped avoid loss. This highlights reserves’ distinct contributions to conservation targets, with private reserves allowing for growth and restoration and public reserves acting as stable anchor points. Our results emphasize the importance of having a diverse array of protected areas to enhance the resilience of reserve networks. We advocate for adaptive regional measures and robust monitoring to achieve global ecological targets.

## Abstract (es)

Efectos del nivel de protección de reservas y la gobernanza sobre la pérdida y ganancia de la cubierta forestal

**Resumen:**

Aunque las áreas protegidas terrestres son esenciales para la conservación de la biodiversidad, todavía no está claro cómo y cuándo los diferentes tipos de áreas protegidas son más efectivos para lograr objetivos específicos de conservación. Evaluamos el impacto de las reservas sobre la pérdida y ganancia de la cobertura forestal a través de un estudio de caso en Tasmania, Australia. Consideramos una variedad de niveles de protección (estricta, en donde están restringidas las actividades humanas, y de usos múltiples) y tipos de gobernanza (pública y privada). Usamos un enfoque de cotejo contrafactual para comparar la pérdida y la ganancia de la cobertura forestal entre las reservas y las áreas desprotegidas cotejadas entre 2004 y 2021. Tomamos en cuenta los cambios en las políticas forestales, las covarianzas ambientales y las presiones humanas para reducir el sesgo de colocación. También caracterizamos a las reservas según su tamaño, gobernanza, manejo y vegetación y comparamos las covarianzas dentro y fuera de las reservas para definir las condiciones de línea base. En general, las reservas establecidas entre 2004 y 2016 tuvieron un 75.4% de menor probabilidad de haber perdido la cobertura forestal y un 16.0% más de probabilidad de haber ganado cobertura forestal en comparación con los controles. Los patrones de pérdida y ganancia variaron según el nivel de protección y el tipo de gobernanza. Las reservas de usos múltiples fueron igual de efectivas que las reservas con actividades humanas restringidas. Las reservas privadas contribuyeron al incremento de la cobertura forestal, mientras que las públicas ayudaron a evitar la pérdida. Esto resalta las diferentes contribuciones que tienen las reservas para los objetivos de conservación, con las reservas privadas que permiten el crecimiento y la restauración y las públicas que funcionan como puntos estables de anclaje. Nuestros resultados destacan la importancia de poseer una gama diversa de áreas protegidas para mejorar la resiliencia de las redes de reservas. Abogamos a favor de las medidas adaptativas regionales y un monitoreo robusto para alcanzar los objetivos ecológicos globales.

## INTRODUCTION

Protected areas have long been the centerpiece of global conservation and will only become more vital given the global target to protect 30% of the earth by 2030. Evaluating their overall efficacy is crucial, but measuring effectiveness is challenging due to the variability in protected area goals, management, location, and design (Rodrigues & Cazalis, 2020). Although current global evidence suggests protected areas can be effective in limiting threats and improving biodiversity, their success in doing so varies significantly (Geldmann et al., 2019; Gray et al., 2016; Gurney et al., 2023). In particular, the different types of protection and governance arrangements have variable impacts depending on context (Ferraro et al., 2013; Geldmann et al., 2019; Oldekop et al., 2016). Understanding the relative impact of these governance arrangements and protection categories is crucial for aligning protected areas with local biodiversity and human needs (Oldekop et al., 2016).

The International Union for Conservation of Nature (IUCN) has 6 protected area categories: strict (Ia–IV) and multiple use (V and VI) (Dudley, 2008). Multiple‐use areas, balancing ecological and socioeconomic goals, are vital for global sustainability targets (Adams, Chauvenet, et al., 2023; Stoudmann et al., 2023). Strictly protected areas prioritize biodiversity conservation with minimal human interference and typically restrict certain activities, such as resource extraction. Globally, strictly protected areas are often located in remote, low‐pressure regions, are biased toward areas with lower economic or agricultural value, and are typically less resourced due to the nature of their governance arrangements (Elleason et al., 2021; Geldmann et al., 2015, 2018). However, few studies compare these categories on a national or regional scale, and those that do report varying outcomes across different contexts. In the Mediterranean, strict protections generally prevent land development more effectively, though exceptions exist (Donnelly & Rodríguez‐Rodríguez, 2022). In Finland, reserve effectiveness on species occupancy depends more on establishment year and size than on IUCN category (Santangeli et al., 2023). In the Brazilian Amazon, the Amazon Protected Areas Program significantly contributes to deforestation reduction in both strictly protected areas and sustainable‐use conservation units (Soares‐Filho et al., 2023). In Acre, Brazil, sustainable‐use protected areas achieve greater deforestation reduction than strictly protected areas due to their placement in high‐threat locations (Pfaff et al., 2014). In Bolivia, Costa Rica, Indonesia, and Thailand, protection strictness and deforestation prevention vary and are influenced by spatial arrangement (Ferraro et al., 2013). This may lead to protection bias toward low‐use areas and thus to protected areas not always aligning with critical conservation needs (Vieira et al., 2019).

Protected areas vary by governance arrangements, which can interact with management categories. Although most are publicly managed, privately managed reserves are significant in regions such as Australia, Southern Africa, and the Americas and are increasingly recognized as key conservation tools (Archibald et al., 2020; Palfrey et al., 2021). These arrangements are often tailored to specific contexts to ensure effectiveness. For example, in Australia, conservation covenants promote tree recruitment and conservation on private land. These covenants involve legal agreements that restrict development and harmful land uses in exchange for financial support, thereby encouraging landowners to maintain and restore natural habitats (Iftekhar et al., 2014; Romanin et al., 2019). Covenants are typically designated as IUCN category IV (habitat and species management area) and focus on specific conservation commitments on individual properties. In contrast, Indigenous protected areas (IPAs), managed by Indigenous communities on a broader scale, integrate traditional knowledge and practices and are generally classified as multiple use (categories V or VI) (UNEP‐WCMC & IUCN, 2024).

Assessments of the impact of these governance arrangements are rare. A recent study focused on conservation covenants in a locality of Victoria, Australia, showed an overall program‐level impact of 0.3–0.8% annual increase in woody vegetation cover and highlighted significant heterogeneity across individual covenants (Sharma et al., 2023). Differences in protected area governance types also affect the conservation of at‐risk vegetation; government and private protected areas show higher representation of these communities compared with IPAs, reflecting historical land tenure allocations (Archibald et al., 2020). These findings underscore the need for context‐specific evaluations to understand nuanced governance effects on conservation outcomes (Palfrey et al., 2021; Zhang et al., 2023).

Protected area impact evaluation studies, including those cited above, have primarily focused on net change in forest area (Ma et al., 2020). However, disaggregating impacts into losses and gains provides a better understanding of how protected areas influence vegetation dynamics. For example, land‐use zoning in Bhutan effectively prevents forest loss but obscures gains in forest cover because regrowth has occurred outside the zoned areas (Bruggeman et al., 2018). In Spain, although total forest area in mountain protected areas has increased only moderately, significant densification of forest stands has occurred, driven by the aggregation of small forest patches (Ameztegui et al., 2021). Targeted reforestation and restoration efforts in multiple‐use and privately managed areas also lead to tree cover gains, particularly where incentives are integrated into management strategies (Sloan, 2016; Ullah, 2024). In southern Africa, protected areas not only reduce deforestation and degradation but also enhance woody growth, resulting in a net increase in aboveground carbon stocks (McNicol et al., 2023). Similarly, on the Tibetan Plateau, effects of protected area edges on vegetation growth vary, highlighting the spatial heterogeneity of conservation efficiency (Hua et al., 2022). This understanding is useful because different protected area designations (in terms of governance arrangements and management categories) often aim to achieve varied ecological outcomes, and without recognizing these distinctions, conservation efforts may not be optimally targeted.

To tailor the design and implementation of protected areas, considering IUCN type and governance arrangements is essential to ensuring these areas deliver desired outcomes (Adams, Chauvenet, et al., 2023). Assessments of reserve networks are vital to the efficacy of conservation efforts because they ensure areas most in need of conservation action are prioritized (Rodrigues et al., 2004). These assessments help build an evidence base to achieve a global understanding of the impacts of reserves and the contextual factors affecting their success (Barber et al., 2012; Hanauer & Canavire‐Bacarreza, 2015). Location‐based analyses, contextually defined based on factors such as policies and land‐use patterns, are needed to build such a global picture. These analyses account for site‐specific factors that may impact the effectiveness of protected areas (Rodrigues & Cazalis, 2020). To contribute to this understanding, we examined how protection levels and governance types at a jurisdiction level affect tree cover dynamics in reserves. We define *effectiveness* as measurable impacts on tree cover, including deforestation prevention and vegetation growth.

The island state of Tasmania, Australia, offers a compelling case study due to its combination of high biodiversity, variety of protected areas, and history of conservation and resource extraction (Law & Kriwoken, 2017; Mackey et al., 2017). Tasmania's situation is reflective of broader global trends, in which conservation areas are often interspersed with lands used for agriculture, forestry, and other human activities, making it an ideal microcosm for studying the complex interactions between protection efforts and environmental change. Our aim was to quantify the impacts of reserves on tree cover loss and gain in Tasmania by considering governance arrangements across publicly and privately managed reserves and IUCN categories. We hypothesized that strictly protected and public conservation areas prevent tree cover loss more effectively and that multiple‐use and private areas show higher tree cover gains due to reforestation efforts. By examining a set of governance types and levels of protection and their specific impacts on tree cover dynamics, we aimed to provide insights that could inform biodiversity conservation strategies in other regions facing similar challenges, thereby contributing to a broader understanding of effective protected area management.

## METHODS

We preregistered our study design on the Open Science Framework (osf.io) before starting the analyses (https://doi.org/10.17605/OSF.IO/VAQHZ) (Parker et al., 2019). All the data we used were freely available. We used QGIS 3.32.2 (QGIS Development Team, 2023) for the spatial analyses. Spatial data were georeferenced to the Geocentric Datum of Australia 1994 (GDA94). All raster data sets not mapped at 25‐m resolution were resampled through bilinear interpolation to this resolution (Table 1). We conducted all statistical modeling in R 4.3.1 (R Core Team, 2023).

**TABLE 1:** Overview of covariates considered and selected based on their relevance to factors influencing tree cover loss and gain or the likelihood of protection, as identified in prior studies and tailored to the ecological and socioeconomic conditions of Tasmania.

Covariate	Description	Unit	Source	Scale
Distance to urban centers	Mean Euclidian distance to nearest urban center	m	Australian Bureau of Statistics—ASGC, 1986 edition	25 m
Distance to reservoir	Mean Euclidian distance to nearest reservoir	m	Own analysis based on Geoscience Australia's Tasmania 1:250,000 GIS data	25 m
Distance to road	Mean Euclidian distance to nearest road	m	Own analysis based on Geoscience Australia's Tasmania 1:250,000 GIS data	25 m
Vegetation type	Main vegetation types; categories: agriculture, urban, and exotic vegetation; dry eucalypt forest and woodland; highland and treeless vegetation; moorland, sedgeland, rushland, and peatland; native grassland; noneucalypt forest and woodland; other natural environments; rainforest and related scrub; saltmarsh and wetland; scrub, heathland, and coastal; wet eucalypt forest and woodland	Categorical	LIST—TASVEG 3.0 groups	1:25,000
Organic carbon content	Organic carbon content within 0–15 cm soil depth; mass fraction of carbon by weight in the <2 mm soil material as determined by dry combustion at 900°C	%	LIST—digital soil maps	80 m
Soil depth	Soil depth (to rooting)	cm	LIST—digital soil maps	30 m
Available water content (AWC)	Available water content at soil depth 0–5 cm	%	LIST—digital soil maps	80 m
Elevation	Average elevation above sea level	m	LIST—digital elevation model	25 m
Slope	Slope (planar angle [0–180])	%	Own analysis based on the LIST—digital elevation model	25 m
Historical rainfall	Multidecadal rainfall (rainfall totals)	mm	Australian Bureau of Meteorology—decadal and multidecadal rainfall	0.01° (approximately 1 km)
Historical temperature	Average annual temperature (mean)	°C	Australian Burean of Meteorology—average annual and monthly maximum, minimum, and mean temperature	0.025° (approximately 2.5 km)

### Case study

Despite its extensive forests and reserves, Tasmania has faced widespread land clearing, mirroring broader Australian trends (Adams, Butt, et al., 2023). The region's history of logging, mining, and agricultural clearing has sparked intense debate about conservation versus resource extraction (Mendel & Kirkpatrick, 2002; Prior et al., 2013). Its forestry sector is a significant player in the global hardwood chip trade and trees are sourced primarily from native forests (Dean et al., 2012). Environmental advocates urge timber harvesting to be limited to designated production forests, whereas the forestry sector seeks access to vast untouched native woodlands. Alongside timber harvesting, the 1970s and 1980s saw widespread clearing for agriculture (Kirkpatrick, 1991).

From the late 1980s, Tasmania adopted a more scientific‐based approach to land management (Mendel & Kirkpatrick, 2002). Tasmania was the last of Australia's states to implement legislation controlling native vegetation clearance when the *Forest Practices Act 1985* was amended in 2002 to prohibit noncommercial clearing of forest for agricultural purposes (Bricknell, 2010). By 2021, 58.7% of native forests were in the state's reserve system (Forest Practices Authority, 2022). Tasmania's reserve system follows the IUCN's protected area categorization: 45% are strict (Ia–IV) and 55% are multiple use (V and VI) (Tasmanian Government, 2023). Multiple‐use reserves hold particular significance in Tasmania, where conflict over native forest timber harvesting continues amid growing global demand and plans to further develop the state's forest industries. Notably, conservation areas and regional reserves prioritize resource provision, including specialized timber harvesting and mineral exploration (Australian Government, 1997).

Tasmania is also experiencing tree cover growth (Potapov et al., 2022). Although tree cover gain in plantation forests commonly indicates rotation cycles, gain outside plantations results from natural recovery following a disturbance, land abandonment, or restoration efforts (Potapov et al., 2022). Socioeconomic and environmental changes since the late 20th century to the present day have led to large expanses of agricultural land being abandoned, allowing for potential natural regrowth (Department of Natural Resources & Environment Tasmania, 2020; Romanin et al., 2019). Fragmented remnants of forests and woody vegetation are scattered across Tasmania's central lowland region, prompting conservationists to advocate for their protection and the establishment of corridors to restore native ecosystems (Davidson et al., 2021). However, remnants are typically small and located on private lands, necessitating alternative governance approaches, such as covenants and other private land conservation programs.

### Study design and temporal focus

We investigated avoided tree cover loss and enabled tree cover gain. We focused on the period from 2004 to 2021 to ensure a consistent policy framework and to leverage the 2002 amendment to the *Forest Practices Act 1985*, which regulates clearing across Tasmania. Selecting 2004 as the starting point allowed us to control for the effects of this legislation and to attribute observed changes in tree cover more confidently to the establishment of reserves rather than variations in forest management policies. The 2003 woody vegetation cover data were not available.

**FIGURE 1:**
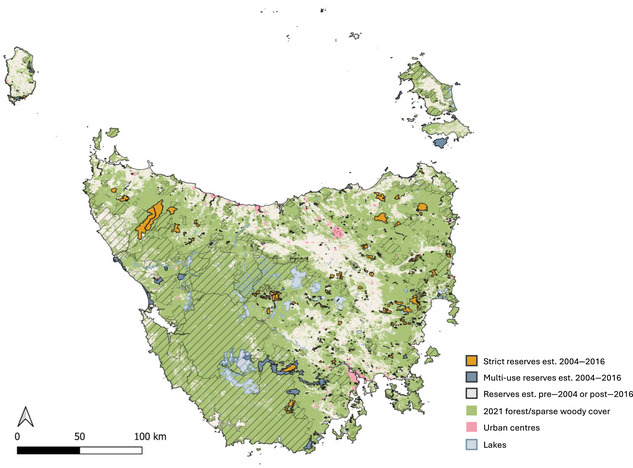
Tasmania's reserve system and woody vegetation cover. The same map with private and public reserve distribution is in Appendix S1.

We sourced reserve data from the Tasmanian Government's Land Information System (the LIST) database that provides a clear distinction between public and private categories and IUCN categories, which we grouped as strict (Ia–IV) and multiple use (V and VI). Reserves established before 2004 were excluded, and control areas were limited to regions never protected prior to 2004 (Figure 1). To ensure sufficient time for the impact of new reserves to manifest, we excluded reserves established after 2016. Our focus on 2 protection levels aligns with other studies in which strict and multiple‐use protection types were compared (Elleason et al., 2021; Ferraro et al., 2013; Huang et al., 2024).

The reasoning behind this design is based on the characteristics of many protected area systems worldwide, including Tasmania. Governments and land conservancies often develop strategic reserve designs to guide property acquisitions over decades (Adams et al., 2011; Miles, 2007). Individual negotiations for purchase or reservation can take many years, with final acquisitions culminating from these extended negotiations. Once acquired, it typically takes several years for a protected area to progress through formal declaration processes (Adams et al., 2011; P. Defourny & M. Appleby, personal communications). During this period, areas often functioned as de facto protected areas before official designation (Ervin et al., 2010; Pitman et al., 2021). For example, a single land sale in 2010 led to the establishment of private covenants from 2010 to 2023 due to organizational time frames needed to repay original founders and finalize covenants (Tasmanian Land Conservancy, 2023). This land purchase resulted in 4 privately protected areas in our data set (declaration periods ranged from 2010 to 2014) and additional covenants established through the revolving fund from 2010 to 2016. Similarly, many public reserves declared during this period were previously forest reserves under protected negotiations for reservation under forest agreements (Australian Government, 2022). Our methodological approach ensured a consistent temporal snapshot in a stable policy setting because the formal declaration of a protected area often culminates from decades of acquisition and management activity not readily observable.

### Data on woody vegetation cover

We used the National Forest and Sparse Woody Vegetation data set for tree cover (Australian Government, 2023), derived from Landsat imagery at 25‐m resolution, available annually for 1988–2021. The data set defines forest (≥20% canopy cover, potentially reaching 2 m high and minimum area of 0.2 ha), sparse wood (5–19% canopy cover), and nonwoody (<5% canopy cover). We reclassified sparse wood to measure woody vegetation presence or absence. We masked plantation forests with the 2021 land‐use layer from the LIST and historic bushfires with the LIST fire history layer to avoid confounding our results because fire can act as a natural disturbance and management tool. Natural disturbances such as insect defoliation and disease were not explicitly distinguished in our analysis due to their patchiness (Schahinger et al., 2003).

### Covariates and spatial data preparation

We collated relevant covariates based on a priori expectations, similar studies (Blackman et al., 2018; Graham et al., 2021; Meng et al., 2023; Schleicher et al., 2017), our regional knowledge, and expert discussions. They covered indicators of human pressure and accessibility (distance to urban centers, reservoirs, roads) and suitability for clearing (soil characteristics, rainfall, temperature, slope, elevation, vegetation type) (Table 1). Vegetation types were quantified using the TASVEG data set, which provides classifications and distributions of dominant vegetation communities across Tasmania at a scale of 1:25,000 (Department of Natural Resources & Environment Tasmania, 2020). To avoid accounting for deforestation due to activity displacement tied to the reserves’ establishment, we excluded units in a zone equal to the size of each reserve (Shen et al., 2022; Zhang et al., 2023). We enforced a minimum distance of 50 m between pixels to reduce spatial dependency (Rahman & Islam, 2021; Shen et al., 2022). By randomly selecting locations in the study region, we aimed to generate an even distribution of sample points across the area (Ferraro et al., 2013).

### Description of reserve characteristics

We characterized the reserves established in the period considered to assess their ecological, geophysical, governance, and management diversity. This characterization involved descriptive statistics of their size, of governance and management type, and of the proportions of dominant vegetation types within their boundaries. We analyzed differences in all covariates considered between areas in‐ and outside the reserve system. This profiling was used to determine the prematching baseline conditions of the study area.

### Statistical matching

Matching allows researchers to reduce differences between treatment and control units to achieve an improved balance in observable covariates (Schleicher et al., 2020). Although more robust alternatives, such as difference‐in‐difference (DiD) and instrumental variable (IV) methods, exist, we used matching for its flexibility and practicality in assessing the causal effect of a single intervention (Meyfroidt, 2016). Matching is widely used in this field, facilitating comparability across different studies (Chen et al., 2023; Ribas et al., 2020).

We used the MatchIt package in R (Ho et al., 2011) to match the treatment units with control units with the most similar covariate values and implemented exact matching on vegetation type. We tested propensity score (PS) and Mahalanobis distances with and without replacement. Our target estimand was the average treatment effect in the treated (ATT). We did not set calipers to ensure representation of all treated subjects (Ho et al., 2011). This decision was based on the equal importance of covariates, negating the need for stricter controls (Andam et al., 2008; Greifer & Stuart, 2021). We chose the matching algorithm that reached the best balance among covariates without discarding any treatment units. The standardized mean difference (SMD), variance ratios, density plots, and love plots in the cobalt package (Greifer, 2023) were used to assess balance (Appendices S2–S4). An SMD <0.25 and variance ratios <2 were considered indications of acceptable balance (Schleicher et al., 2020; Stuart et al., 2013) (Appendix S5). Our final sample sizes were from 8% to 18% of treatment pixels, balancing computing time and robustness. The control sample size for the loss analyses was 4,748,725 pixels, and for the gain analyses, it was 3,438,956 pixels. Mahalanobis distance outperformed PS distance in all but one case. All matching analyses were performed without replacement. The sample size of each treatment group and the final matching specifications are outlined in Appendix S6.

### Effect estimate

We used a logistic regression model with the glm function and applied a binomial logit link with treatment–covariate interactions. Specifically, our response variable was the binary outcome of tree cover change (gain or loss). The main effects of the treatment and the interaction effects between the treatment and each covariate were included. The marginal effect was estimated as the coefficient in the outcome model, and standard errors (SEs) and confidence intervals (CIs) were estimated using cluster‐robust SEs. The estimand ATT was calculated by performing g‐computation with the avg_comparison function from the marginaleffects package (Arel‐Bundock, 2023), and the effect was measured by marginal odds ratio (OR). We calculated the percentage of avoided loss and enabled gain per reserve type by dividing the average woody vegetation cover loss or gain rate of the control units by the average loss or gain rate in the treatment units, adjusting for whichever rate was higher.

To analyze the sensitivity of our results, we used the evalue package (Linden et al., 2020) to calculate the *E* value for the OR of each analysis. Although sensitivity analyses to hidden bias cannot explicitly reveal presence of hidden bias, *E* values show the strength that a potential confounding effect would need to have to negate an observed treatment effect (Van Der Weele & Ding, 2017). A high *E* value suggests that a substantial amount of unmeasured confounding would be required to account for an effect estimate, and the lowest *E* value of 1 indicates that no unmeasured confounding would be necessary to explain away the association. Additionally, we tested the sensitivity of results to different model specifications by estimating the treatment effect based on various matching algorithms, as outlined above.

## RESULTS

### Characteristics of reserves established from 2004 to 2016

**FIGURE 2:**
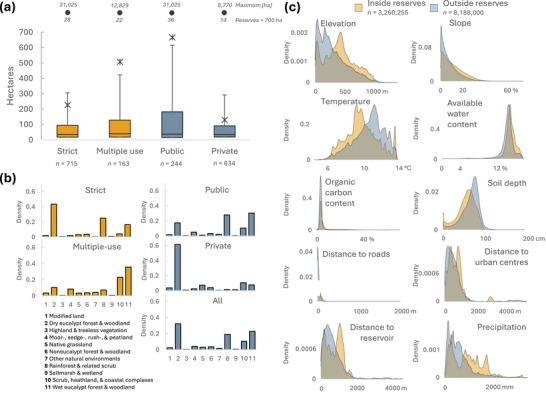
(a) Size distributions of terrestrial reserves, as designated by the International Union for Conservation of Nature, established from 2004 to 2016 (*n*, number of reserves; reserves larger than 700 ha not displayed for readability, but their count and maximum size are provided above the box plot), (b) density of vegetation types across reserve units, and (c) density of covariates across reserve units (yellow curves) and a sample of units outside reserves (blue curves). Production forests and historically burned areas are masked.

In total, 878 reserves spanning 244,790 ha met our inclusion criteria. Of these, 715 were under a strict category of protection,163 were multiple use, 244 were public, and 634 were private. The median sizes of strict, multiple use, publicly managed, and privately managed reserves were relatively similar, ranging from 34 to 40 ha. Publicly managed reserves had a mean size of 666 ha, privately managed reserves averaged 130 ha, multiple‐use reserves had a mean size of 508 ha, and strict reserves had a mean of 227 ha (Figure 2).

Dominant vegetation types in these reserves also differed between groups (Figure 2). Strict reserves showed similar patterns across all reserves, and the 3 forest vegetation types (dry eucalypt, rainforest, and wet eucalypt) were most represented. Multiple‐use reserves were mostly home to wet eucalypt and scrub and heathland and coastal complexes. When grouped by governance type, public reserves had similar representation as strict reserves, represented mostly by the 3 fully forested vegetation types. In contrast, 61.4% of privately managed reserves were in dry eucalypt vegetation types.

Covariate values inside and outside reserves exhibited notable differences (Figure 2). Reserve units (including tree‐covered and nontree‐covered area), on average, were situated at higher elevations (423.9 vs. 315.8 m) and on steeper slopes (11.2 vs. 8.0%), were positioned farther away from roads (49.9 vs. 30.3 m), urban centers (733.0 vs. 650.2 m), and reservoirs (769.1 vs. 687.3 m), and received a higher annual precipitation (1210 vs. 964 mm) and comparatively lower temperatures (9.9 vs. 10.7°C) compared with areas outside the reserves. Reserves had higher soil organic carbon (SOC) content (9.1% vs. 6.2%) and shallower soil depth (55.0 vs. 65.2 cm). Available water content (AWC) in soil (13.2% vs. 12.8%) was the only covariate with similar average values in‐ and outside reserves. Pre‐ and postmatching density graphs for the samples used in each analysis are in Appendix S4.

Regarding the reserve area distribution, 66.4% was publicly managed, of which 54.7% and 45.3% was dedicated to strict and multiple‐use reserves, respectively. Private areas accounted for 33.6% of the protected area, of which 89.0% fell under the category of strict, all of which were conservation covenants, and 11.0% fell under multiple use, made up of 2 IPAs. Overall, strict reserves represented 66.2% of the total area protected, whereas multiple‐use reserves constituted 33.8%.

### Effect estimate for tree cover loss

**FIGURE 3:**
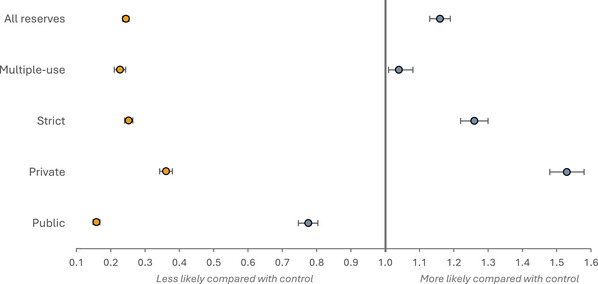
Odds ratios and their lower (2.5%) and upper (97.5%) confidence intervals for the effects of different protection levels (strict and multiple use) and management types (public and private) on tree cover loss and gain.

Considering 2021 woody vegetation cover, reserves established from 2004 to 2016 led to a decrease in the likelihood of woody vegetation loss compared with areas outside reserves, regardless of protection level and governance type (Figure 3). Across all reserve units, the odds of woody vegetation in reserves being lost was 0.25 times less (i.e., 75% lower) than for woody vegetation outside reserves. There was little variation between strict and multiple‐use reserves (ORs of 0.25 and 0.23, respectively). When grouping these sample reserves by governance type rather than level of protection, public reserve units showed to have decreased the odds of experiencing loss by 84% (odds of 0.16), and the odds of privately managed reserve units were 0.36 compared with the control. The *E* values computed per analysis were 4.7–11.2, suggesting relatively robust estimates (Appendix S6).

### Effect estimate for tree cover gain

Although the odds of tree growth occurring in 2021 inside reserves were 1.2 compared with outside reserves (i.e., 20% more likely), the effect was heterogeneous across subgroups (Figure 3). Strict reserves were more likely than multiple‐use areas to have experienced growth compared with their respective controls; strict reserve units had odds of 1.3 and multiple‐use reserves had odds of 1.04 compared with outside reserves. Considering governance types, private reserve units were 53% more likely than their control sample to experience tree growth. In contrast, public reserves were less likely than similar areas outside reserves to have had growth on land without woody vegetation cover. In contrast to the loss analyses, the gain estimates showed comparatively weaker confounder associations; *E* values ranged from 1.8 to 1.2 (Appendix S6).

### Avoided loss and enabled gain

**FIGURE 4:**
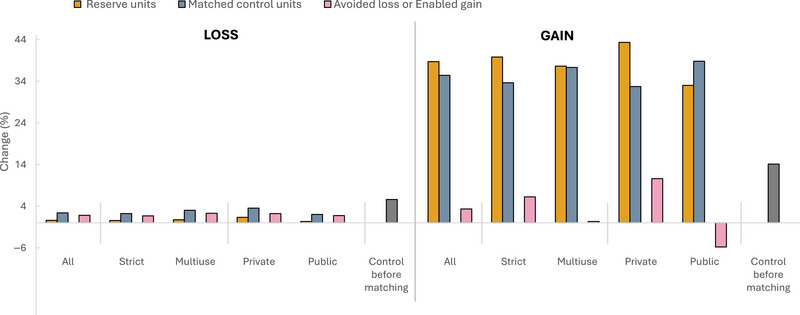
Percentage change in woody vegetation cover in treatment reserve units, matched control units, and all control units before matching. Avoided loss or enabled gain represents the difference between rates of change in reserve and treatment units.

Across all reserves, from 2004 to 2021, 0.6% of the treatment sample (i.e., units in reserves with woody vegetation cover) lost woody cover, versus 2.4% in the control sample, meaning that the presence of reserves helped avoid 1.8% loss in reserves (Figure 4). Strict reserves avoided 1.6% loss (0.6% loss in reserves vs. 2.2% in control) compared with 2.3% in multiple‐use reserves (0.7% inside vs. 3.0% in control). More control units experienced loss compared with treatment units in privately managed reserves (3.5% vs. 1.3%, respectively), representing the highest percentage across control groups and an avoided loss of 2.2%. Public reserve units had the least loss (0.3%) relative to their control group (2.0%) (i.e., 1.7% avoided loss). Previously, areas without woody vegetation cover experienced high levels of tree cover growth inside and outside reserves. Enabled tree cover gain was highest in strict and privately managed reserves in both time periods. In reserves without woody cover, 6.2% more units gained tree cover compared with control units from 2004 to 2021 in the former, and 10.6% gained tree cover in the latter. For multiple‐use reserves, only 0.3% more units gained tree cover compared with control. Public reserves were the only case in which units inside experienced less gain than control; control units experienced 5.8% less gain than control units.

## DISCUSSION

Our findings highlight the important role of reserves in mitigating tree cover loss and promoting tree cover gain and reveal variability in their effectiveness based on protection level and governance type. Our results can inform conservation strategies in other regions with similar ecological and socioeconomic contexts. The reserves included in our study were established following the 2002 amendment of the *Forest Practices Act*, imposing stricter regulations on clearing across Tasmania. On average, these reserves were established in areas with fewer human pressures and less agricultural suitability than nonreserve areas. Through statistical matching, we controlled for this nonrandom allocation across the landscape. Areas in reserves were significantly less likely to be cleared compared with areas outside and more likely to have experienced tree cover growth. However, distinct patterns emerged when considering the subgroup analyses by level of protection or governance type.

Our results add to the body of evidence showing that multiple‐use protected areas can effectively contribute to biodiversity conservation (Adams, Chauvenet, et al., 2023; Geldmann et al., 2018). Multiple‐use reserves avoided a similar share of tree cover loss compared with strict reserves. Similar studies provide examples of cases in which stricter areas were more effective than multiple‐use ones (Nelson & Chomitz, 2011; Shah et al., 2021). Other studies showed results consistent with ours: little difference in effectiveness (Ferraro et al., 2013; Paiva et al., 2015). Regional variability in effectiveness can be driven by context‐specific factors, such as management quality and socioeconomic conditions (Rodrigues & Cazalis, 2020; Shah et al., 2021).

We found multiple‐use units to only be slightly more likely than control units to experience growth, whereas strict reserves had a significantly higher likelihood of growth compared with controls. In Tasmania, activities allowed in multiple‐use reserves may explain the observed differences. Conservation areas and regional reserves make up 210 of the 878 reserves established from 2004 to 2016, and they prioritize timber and mineral resource provision (Australian Government, 1997). Although non‐native production forests were excluded from our analysis, certain areas in these multiple‐use reserves likely experience extractive activities that inhibit patterns of tree cover gain from occurring while avoiding excessive loss of existing woody vegetation. Conversely, strict protection limits disturbance and thus allows for more vegetation growth and limiting loss.

We found a significant difference in the effectiveness of publicly and privately managed reserves. Public reserves were more effective in preventing tree cover loss, and privately managed reserves were more likely to experience tree growth in previously nonwoody areas. These results likely reflect the differing objectives and management practices of each governance type, as well as the motivations and property types each reflects. Conservation covenants, comprising most of the privately managed reserved area in our study, were often established on land previously subjected to intensive use, necessitating regeneration or restoration. In Tasmania, many covenants are in the Midlands, the island's main agricultural region and a focal point of conservation efforts (Gilfedder et al., 2021). These reserves are established not only to protect threatened ecosystems but also to restore them, leading to greater tree growth potential compared with public reserves, which often target areas that are relatively intact (Fitzsimons & Carr, 2014; Hardy et al., 2017). The distinct roles of conservation covenants and public reserves in biodiversity conservation reflect their respective management objectives and the landscapes they occupy. Conservation covenants can act as dynamic networks that reduce fragmentation and contribute to a more resilient reserve system (Palfrey et al., 2021). Incorporating these covenants in national and regional conservation strategies can also help address gaps in the global protected area network because many regions with high conservation value are underrepresented (Jenkins et al., 2013; Rodrigues et al., 2004). Our findings highlight the need for tailored management approaches for different reserve types, a topic that has not been extensively explored in the literature.

Differences in management tools used in private and public reserves may also explain our results, particularly the application of fire. Although we masked historical fire scars, smaller fire occurrences may have been included. In conservation covenants, controlled burning is strongly avoided by landholders, unlike in public reserves where it is more commonly used (Halliday et al., 2012). This avoidance could lead to more thickening of the vegetation in privately managed reserves compared with public ones. Additionally, our sample showed that a greater share of publicly managed treatment areas had tree cover at the baseline compared with treatment areas in private reserves. This indicates that public reserves, often located in relatively intact habitats, had less potential for additional tree growth because they were already woody. Conversely, private reserves, established in previously degraded or intensively used areas, exhibited higher potential for tree growth because they underwent restoration efforts and regeneration.

Our results align with a recent study that showed covenants in the Goldfields region of Victoria, Australia, led to increased woody vegetation, though yearly variations were observed (Sharma et al., 2023). Although our results were positive, there is high variability in the objectives and in the monitoring and evaluation of private reserves, making measuring their effectiveness difficult (Fitzsimons & Carr, 2014; Zhang et al., 2023). Nevertheless, more resources need to go toward monitoring because conservation covenants are long‐lasting, permanent interventions in the landscape (Hardy et al., 2017; Selinske et al., 2019).

Reserves represent static boundaries in a fast‐changing environment. Dynamic strategies should be implemented to ensure resilience, such as incorporating biodiversity patterns into expansion plans and protecting transitioning areas (D'Aloia et al., 2019; Dobrowski et al., 2021). Our results showed that although public reserves serve the role of stable nodes, by helping to avoid loss but not experiencing more gain than controls, private reserves provide opportunities for restoration and potentially for establishing linking corridors. Privately managed reserves are an important tool to create biodiversity networks, particularly across high‐pressure working landscapes and culturally significant areas (Davidson et al., 2021; Tasmanian Aboriginal Centre Inc., 2015). Improved monitoring and impact evaluations are essential to ensure that limited resources are spent efficiently and that positive outcomes for biodiversity are maximized (Fitzsimons & Carr, 2014).

Several caveats concerning our results deserve mention. Although we assumed tree cover leads to positive biodiversity outcomes, this is not necessarily the case (Veldman et al., 2015). Other threats affecting reserves’ effectiveness are not observable through spatial analyses (Schulze et al., 2018), meaning our outcome variable may not fully depict on‐ground reality. Additionally, although our covariates captured the environmental conditions, we did not include landscape context covariates, such as proportion of woody vegetation cover around reserves, which may provide more information on how surrounding land use influences vegetation dynamics. Our choice of statistical approach influenced the robustness of our findings. Matching focuses on pretreatment covariates to control for observable confounders but may not address unobservable factors. Other methods, such as DiD or IV, could offer insights by leveraging temporal comparisons, potentially revealing dynamic treatment effects or mitigating unobserved heterogeneity. Finally, the computed *E* values indicated that although the effect estimates of the loss analyses remained robust to hidden biases, the gain analyses may have been more susceptible to unobserved confounding factors. Our planned next step is to assess conservation covenants at the local level to better understand the causal mechanisms underpinning our findings.

Our results showed the critical role of reserves in mitigating tree cover loss and fostering tree cover gain and that effectiveness varied based on protection level and governance type. Although multiple‐use reserves exhibited comparable effectiveness to strict reserves, distinct management strategies emerged between publicly and privately managed reserves, each playing unique roles in promoting biodiversity conservation and restoration. The mixed effectiveness of public and private reserves highlights the importance of a diversified conservation strategy that capitalizes on the strengths of different governance types. This approach is relevant for regions with fragmented landscapes and varying land ownership patterns. Drawing on the lessons learned from Tasmania, conservation practitioners and policymakers can develop more robust and context‐specific strategies that enhance the resilience and ecological integrity of protected areas globally and that contribute to growing evidence and monitoring of area‐based conservation interventions. We found that although all the area‐based conservation approaches we examined (strict, multiple use, public, and private) had an effect, each performed better in a specific context. Understanding these contexts is crucial to choosing the best type of protection for local objectives, particularly as conservationists plan for how to protect, conserve, restore, and sustainably use areas to meet targets. Through our comparative analysis, we identified the strengths of different governance types and levels of protection. This information can help decision makers choose the conservation strategies that will best meet conservation goals.

## Supporting information



Additional supporting information may be found in the online version of the article at the publisher's website.
Supplementary Information
