# Supplementary material for: Effect of reserve protection level and governance on tree cover loss and gain
Source: Conserv Biol. 2025 Jan 24;39(4):e14449. doi: 10.1111/cobi.14449 (PMC12309654; doi:10.1111/cobi.14449)
Supplement: Supplementary file 1 — Additional supporting information may be found in the online version of the article at the publisher's website. Supplementary Information [file COBI-39-e14449-s001.docx]

# **SUPPLEMENTARY INFORMATION**

Effect of reserve protection level and governance on tree cover loss and gain

**Natasha Stoudmann ^1^, Jason Byrne ^1^, Vanessa Adams ^1^**

^1^ School of Geography, Planning, and Spatial Sciences, University of Tasmania, Hobart, TAS, Australia

**Appendix S1:** Public and private reserves included in the study.


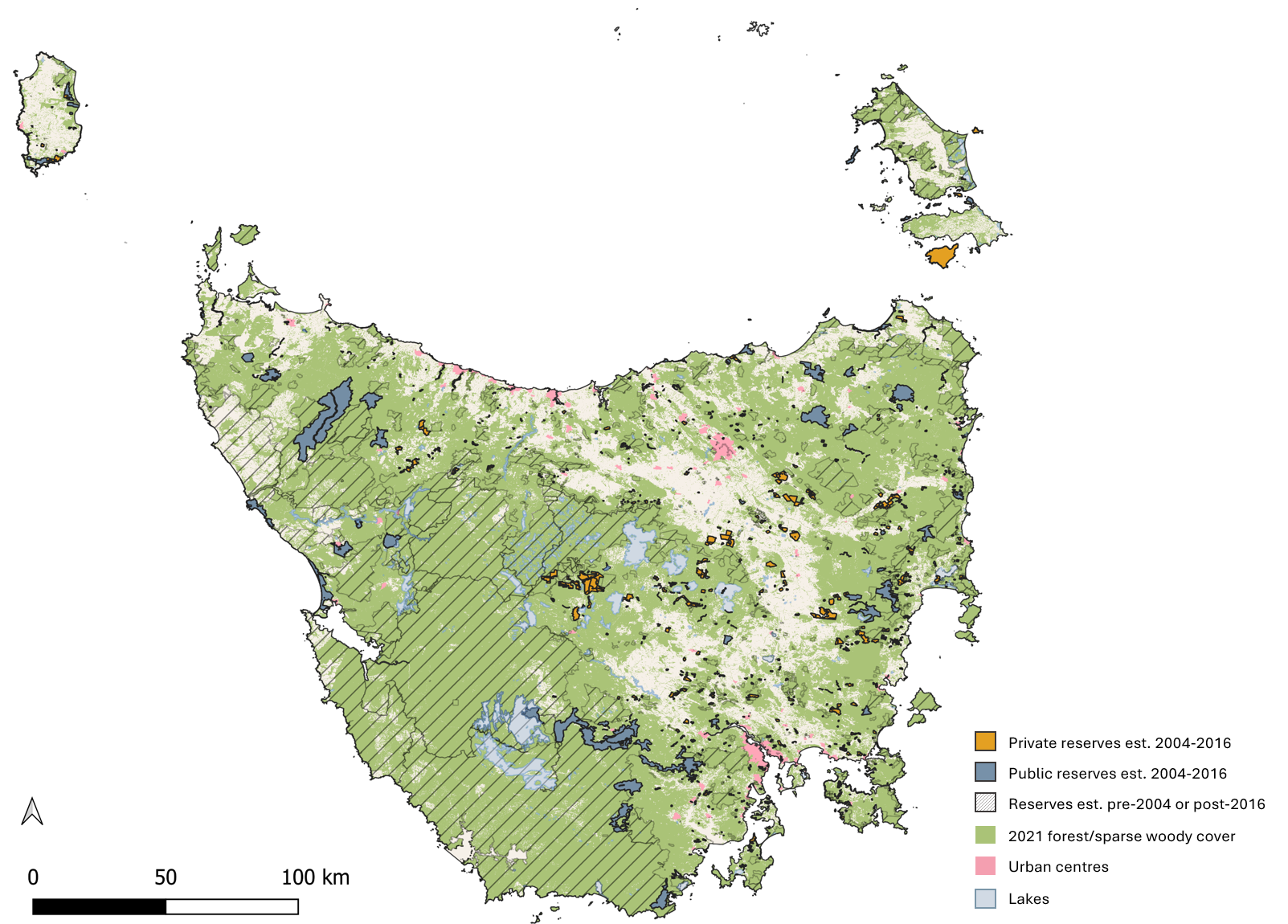


**Appendix S2**: Balance plot of each analysis, showing the standardised mean difference of covariates before and after matching.


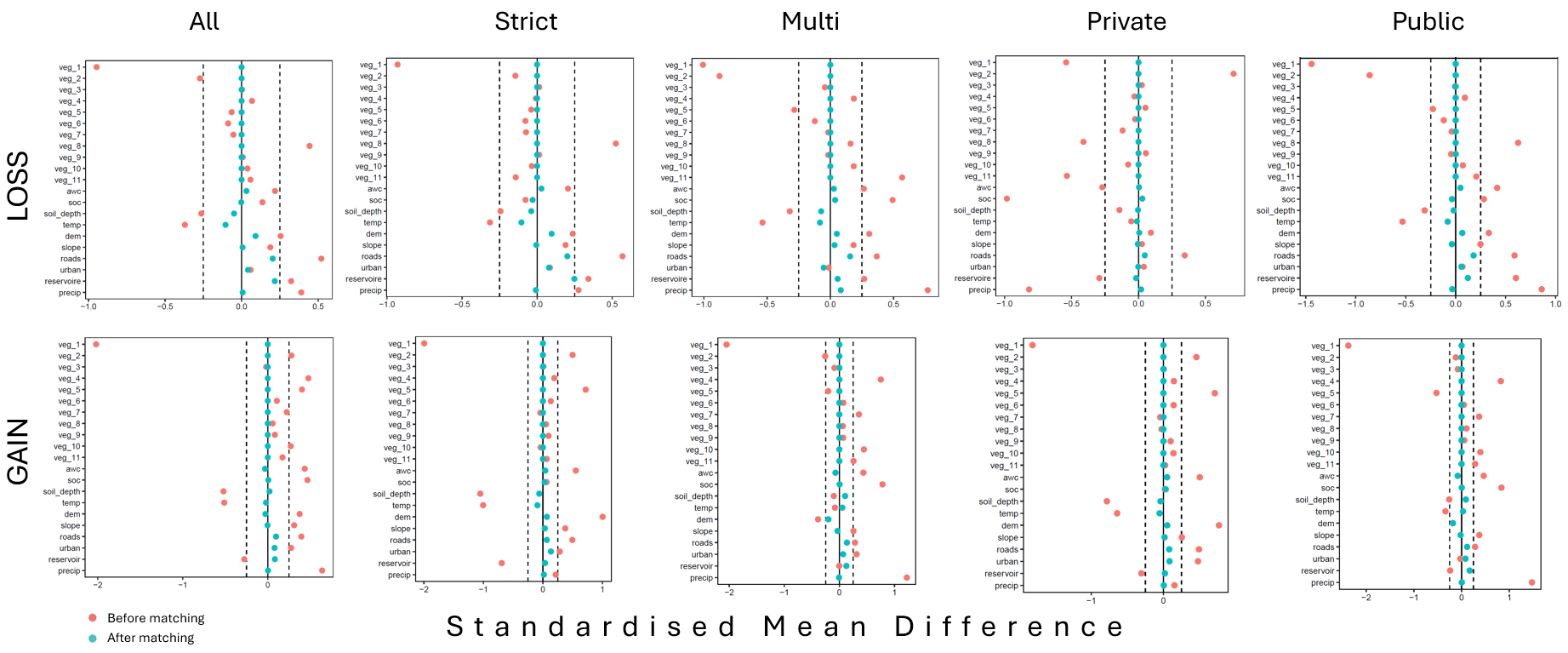


**Appendix S3:** Density curves of all covariates before and after matching for all tree cover loss analyses.


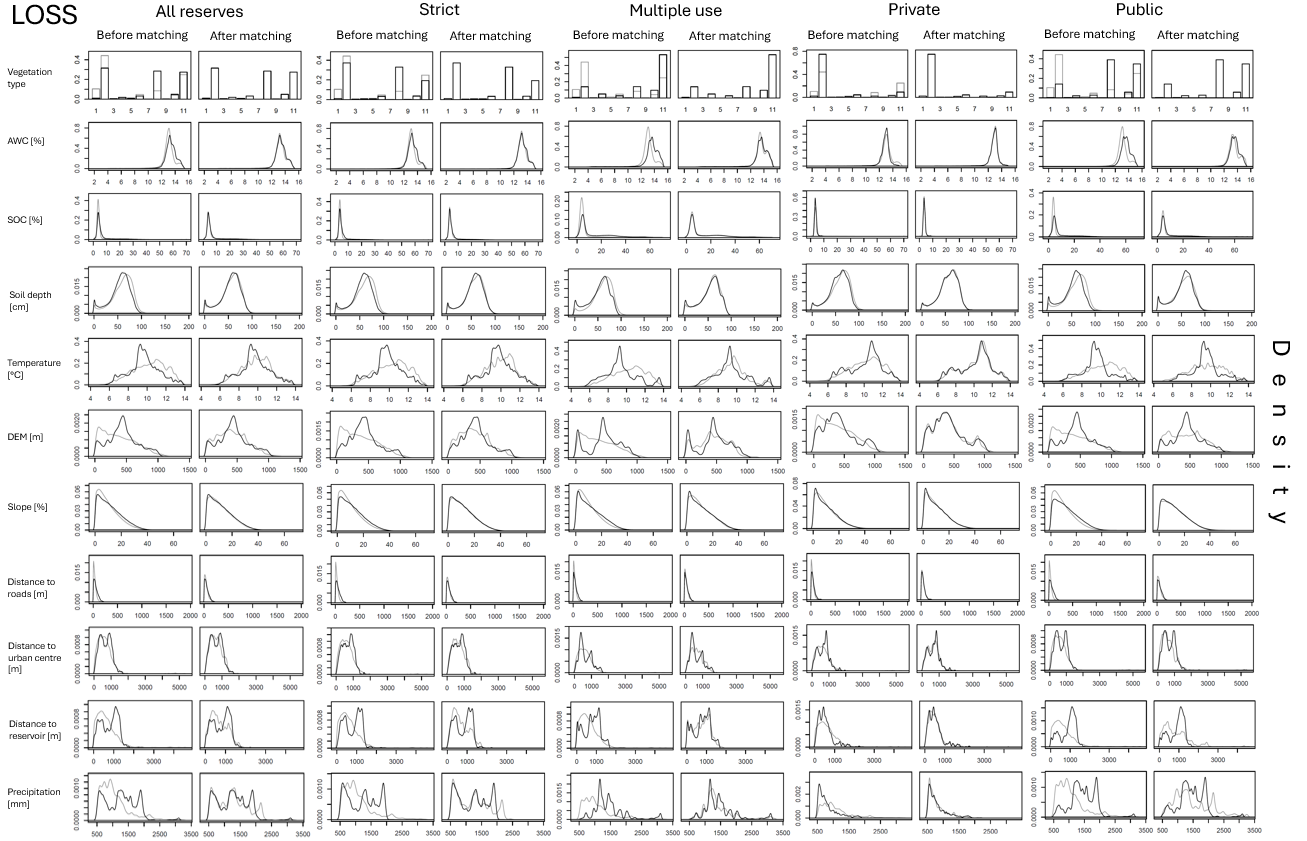


**Appendix S4:** Density curves of all covariates before and after matching for all tree cover gain analyses.


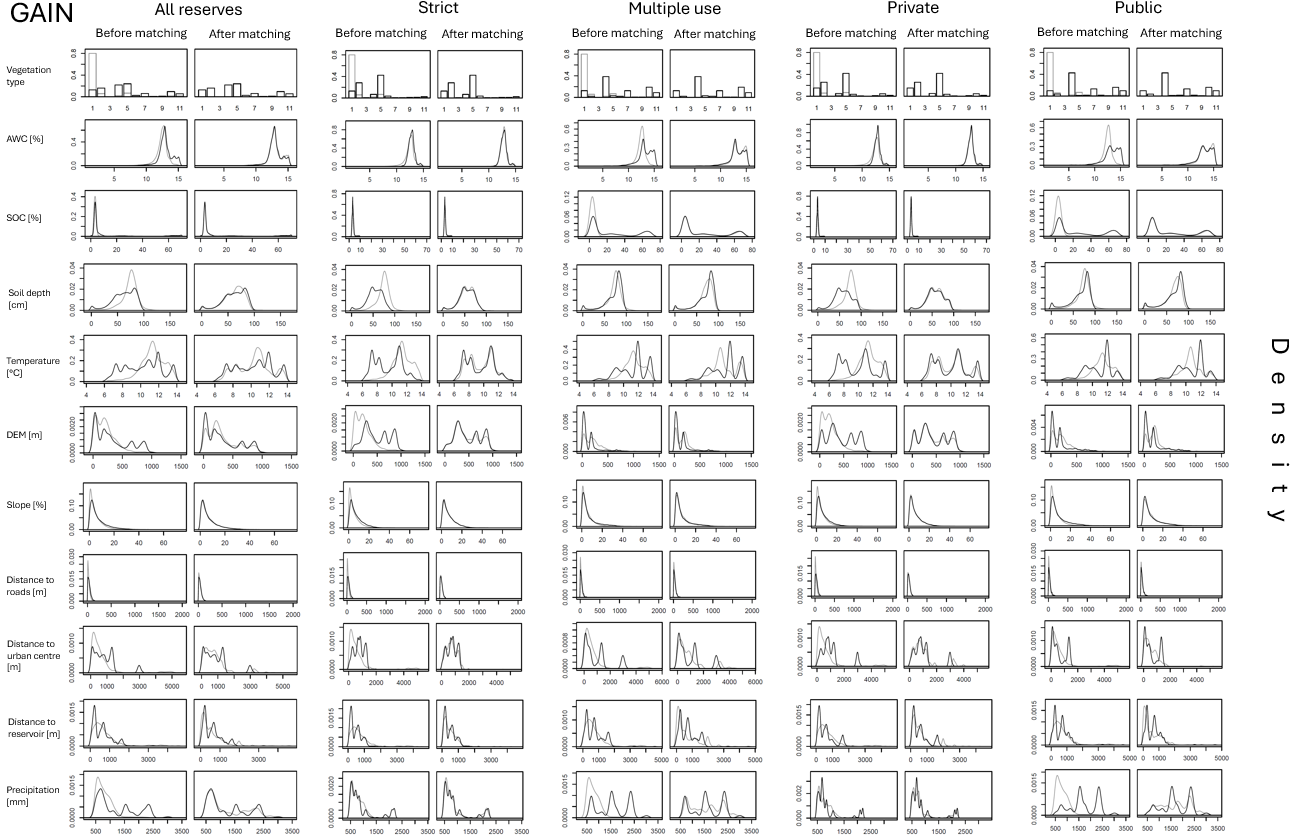


**Appendix S5:** Summary of balance per analysis, before and after matching.

**Loss – All reserves**

**
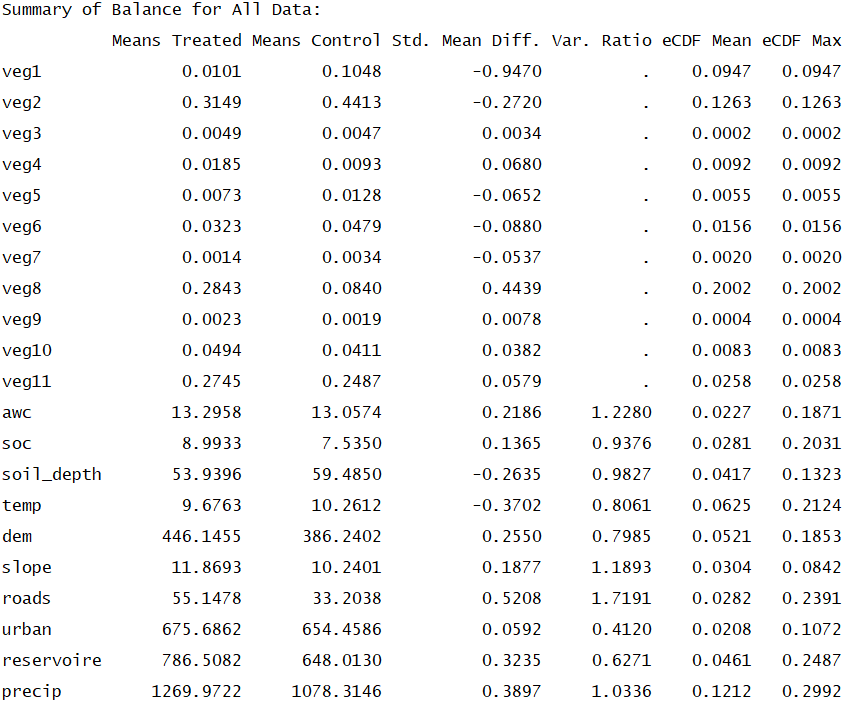
**

**
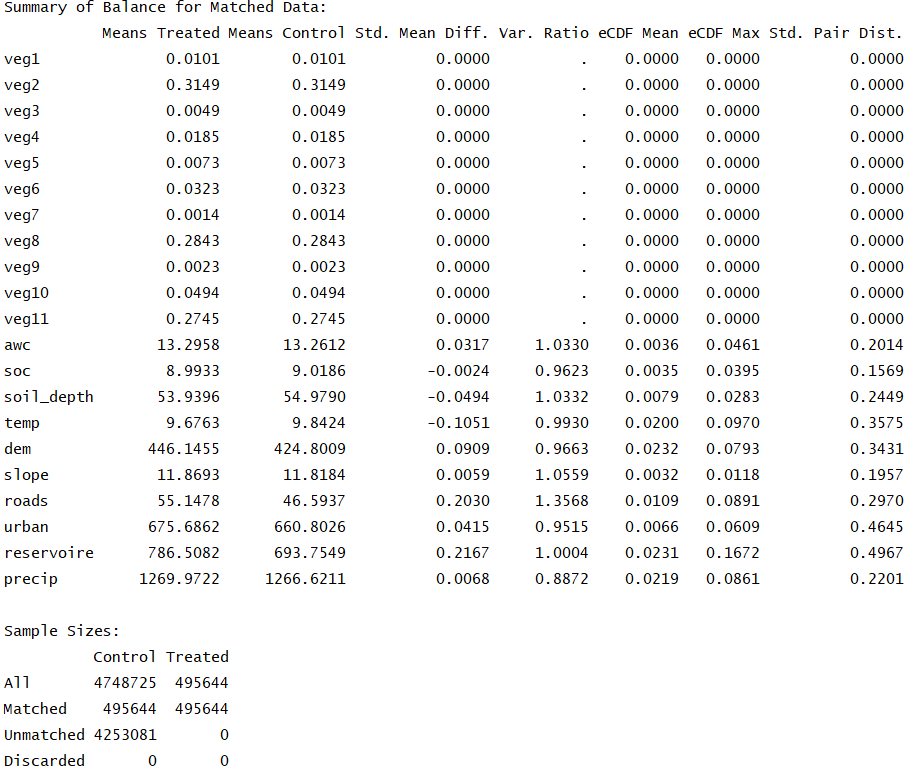
**

**Loss – Strict reserves**

**
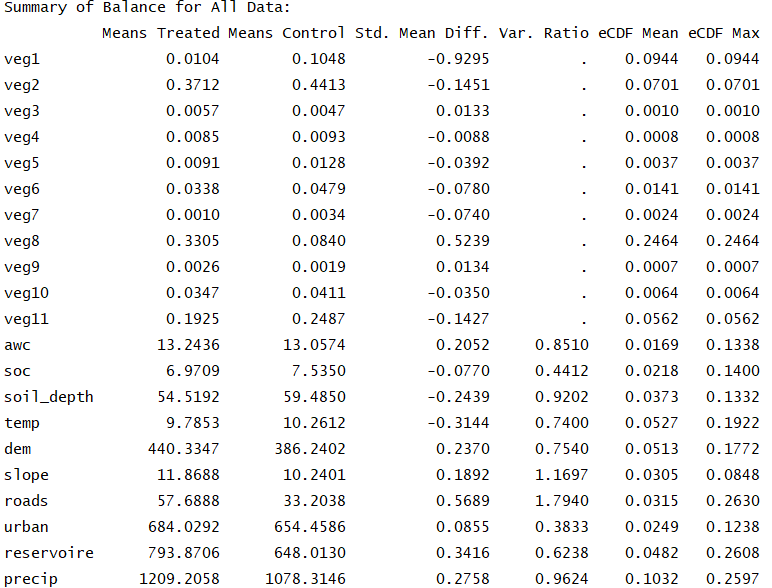
**

**
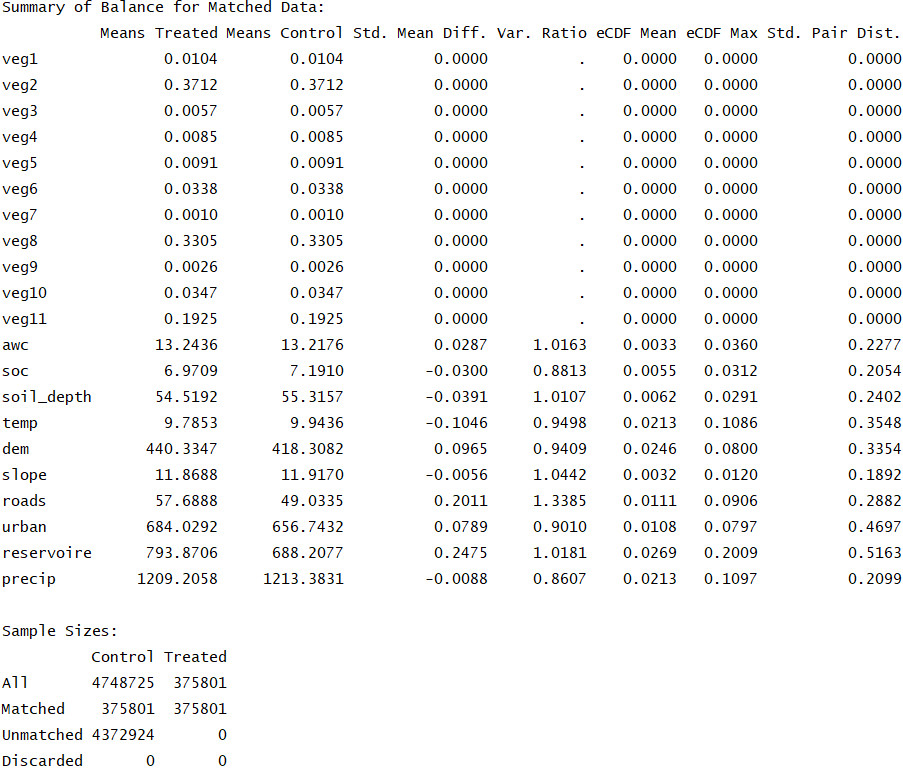
**

**Loss – Multiple use**

**
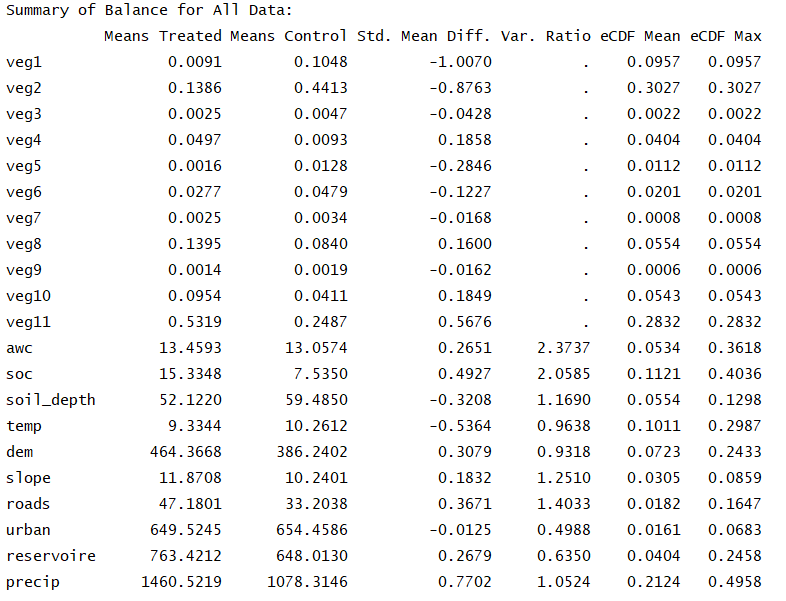
**

**
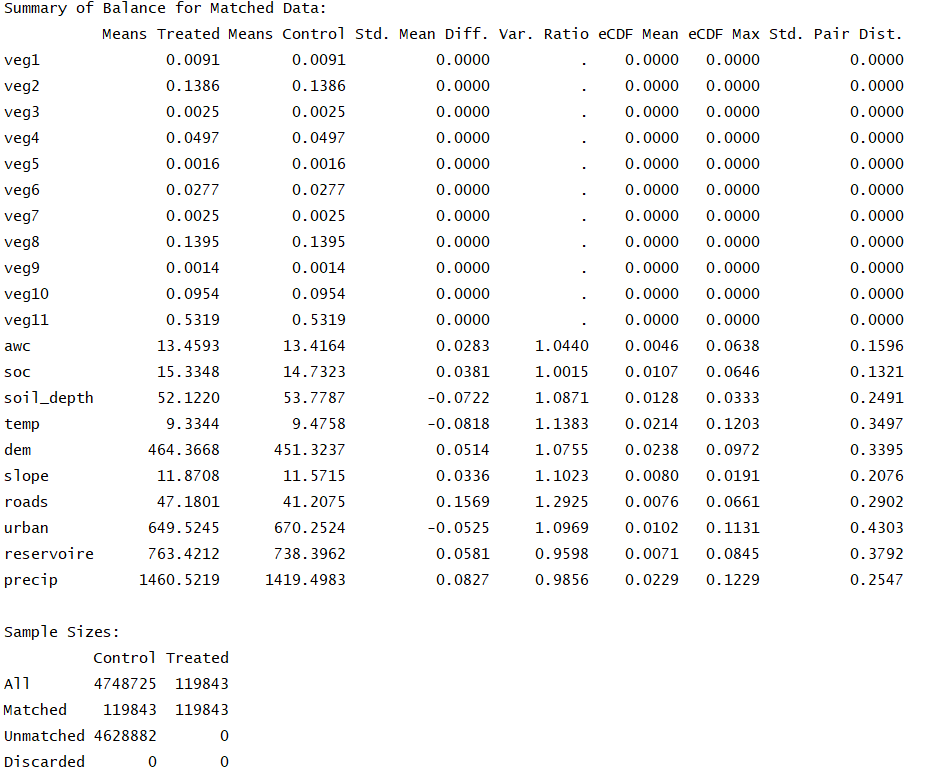
**

**Loss – Private reserves**

**
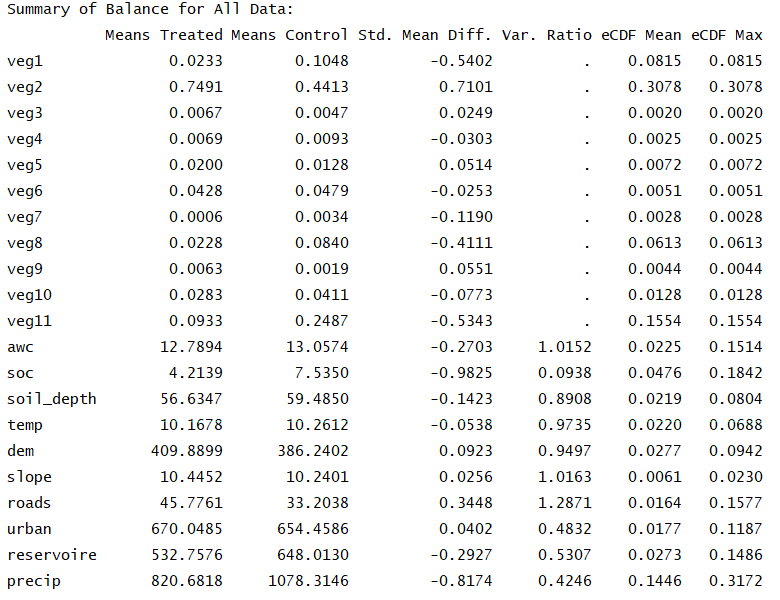
**

**
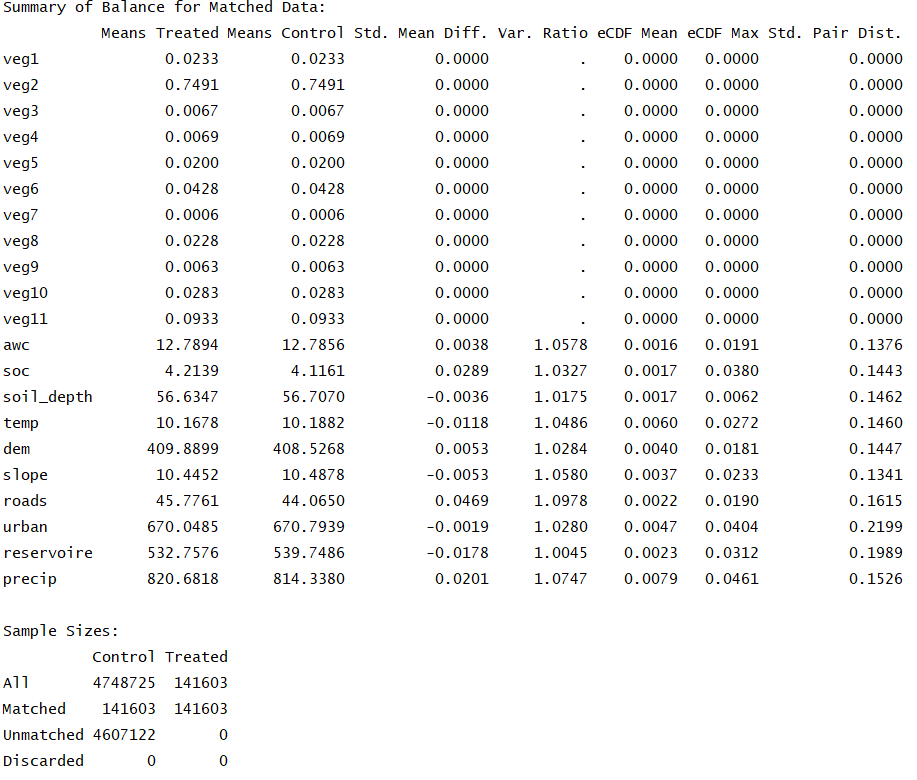
**

**Loss – Public reserves**

**
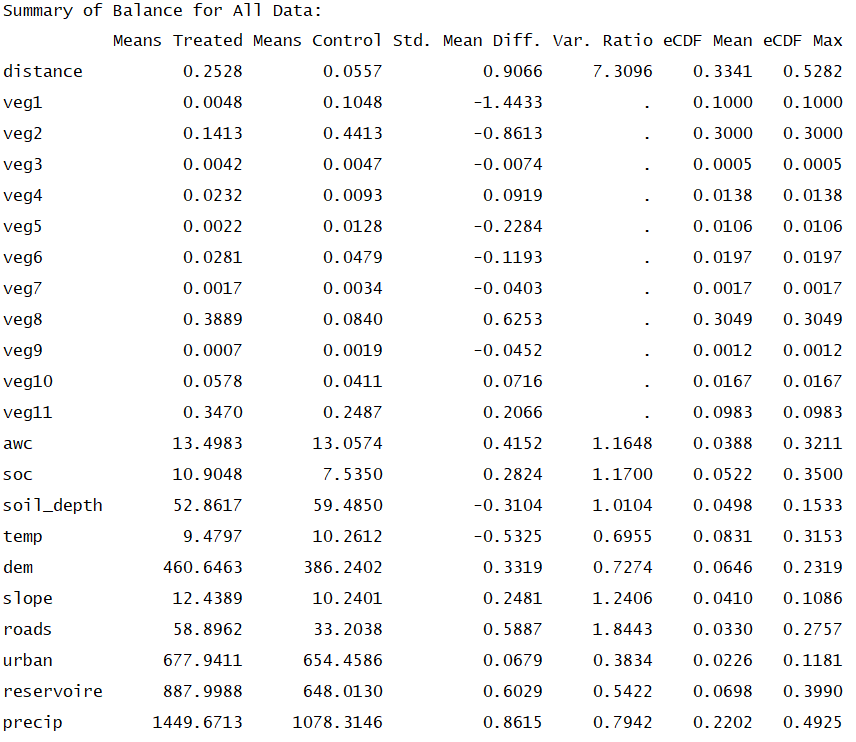
**

**
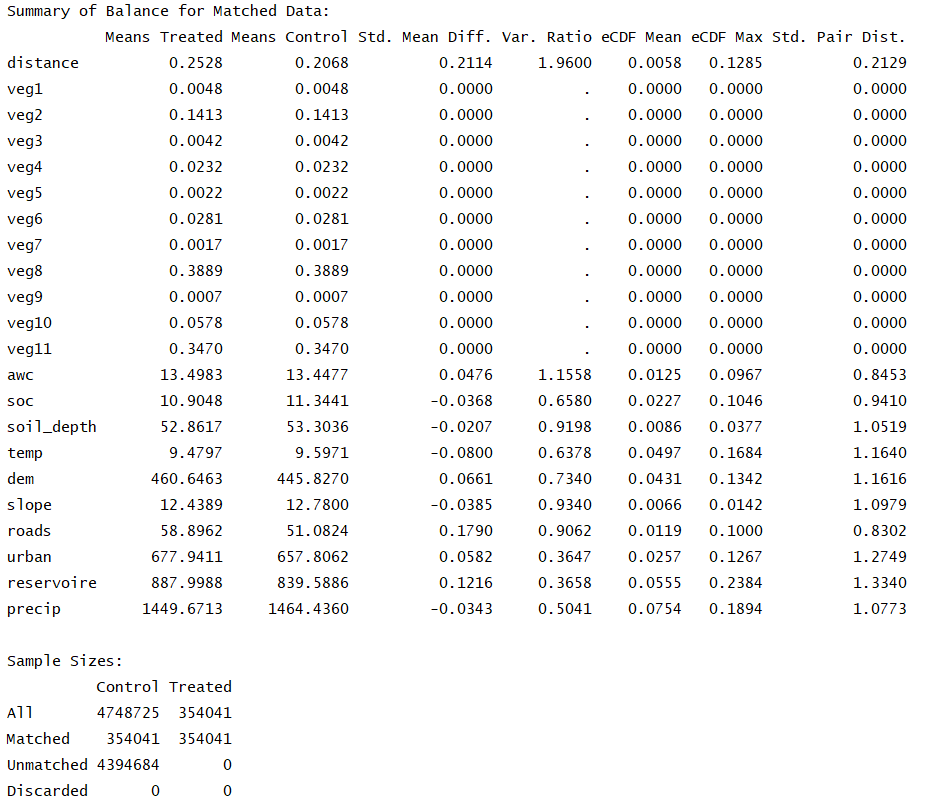
**

**Gain – All reserves**

**
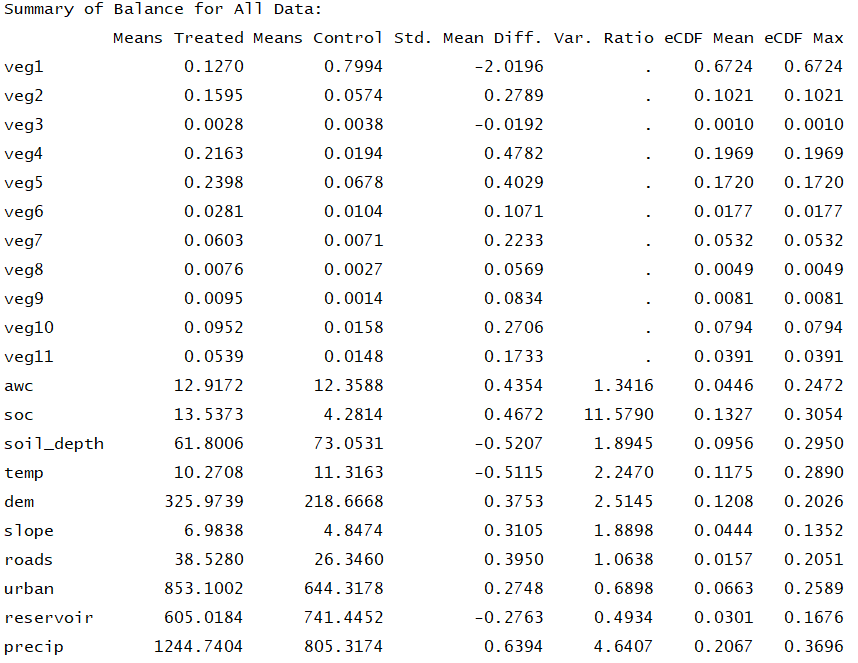
**

**
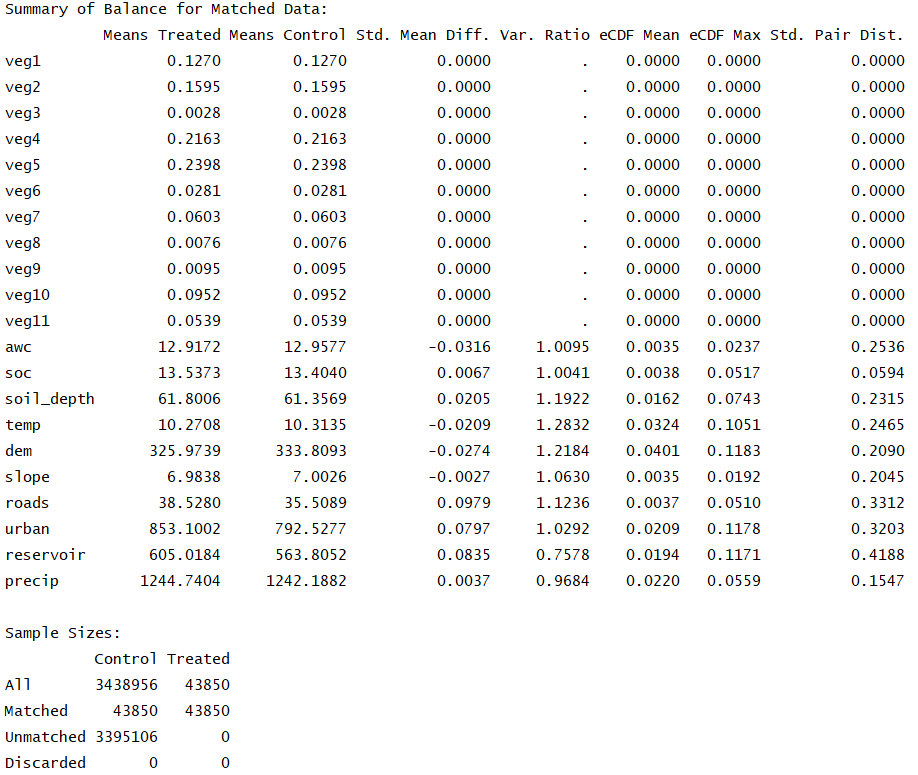
**

**Gain – Strict reserves**

**
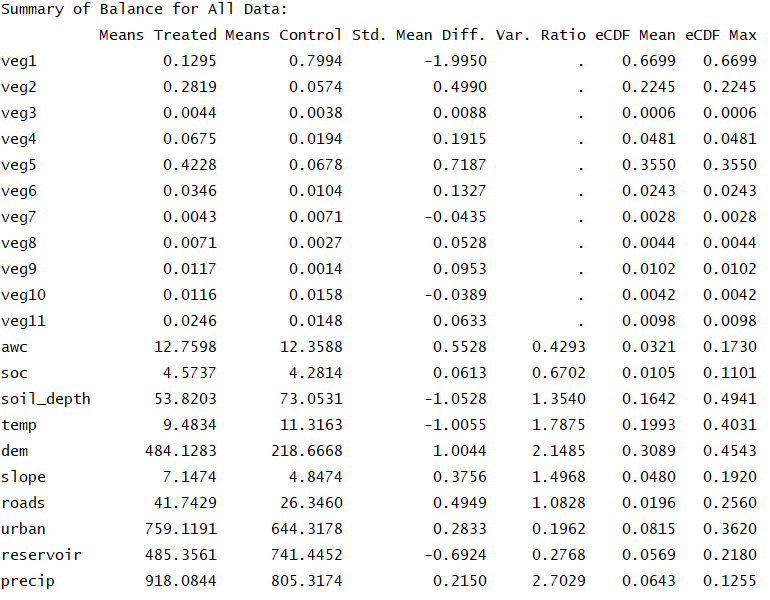
**

**
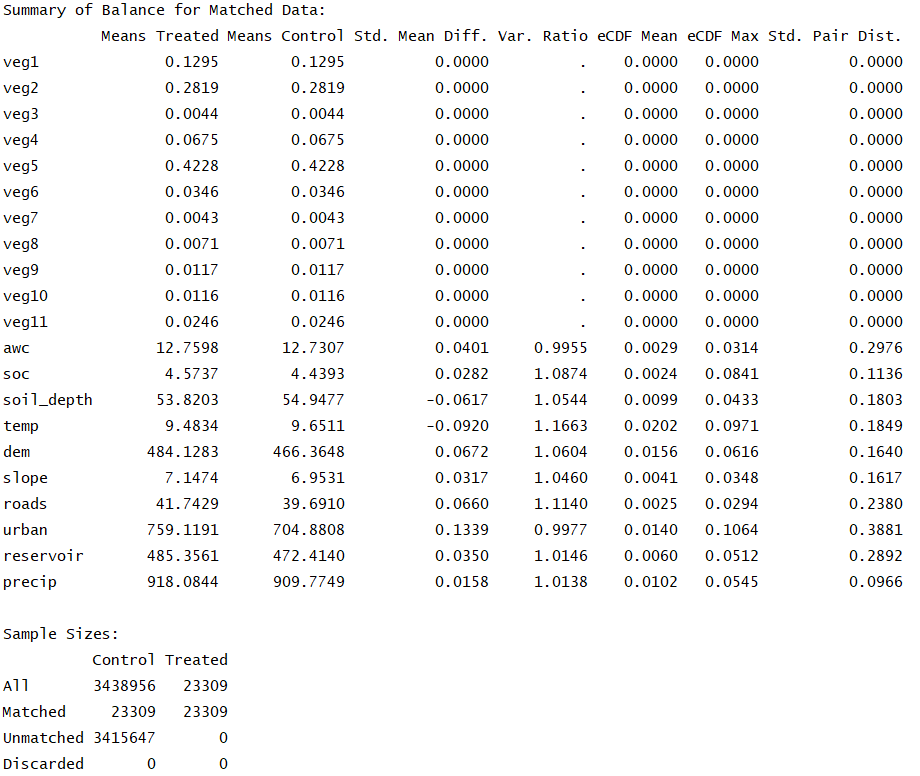
**

**Gain – Multiple use**

**
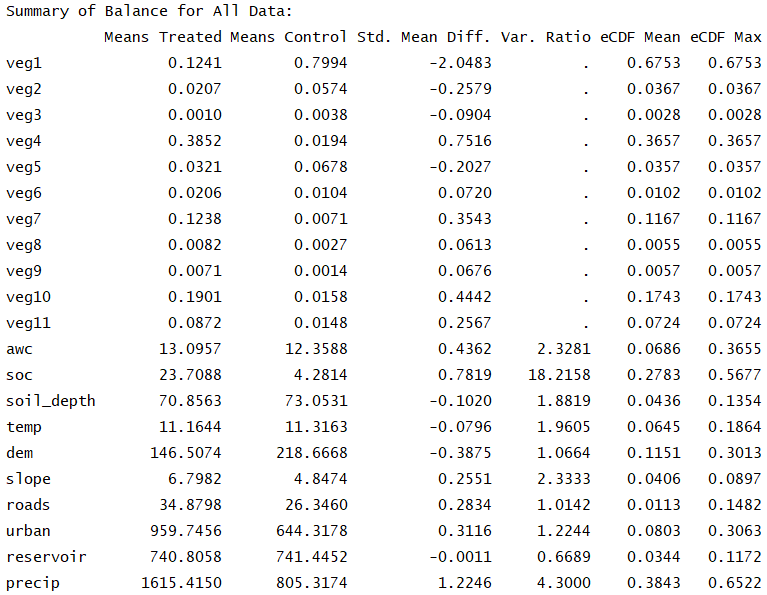
**

**
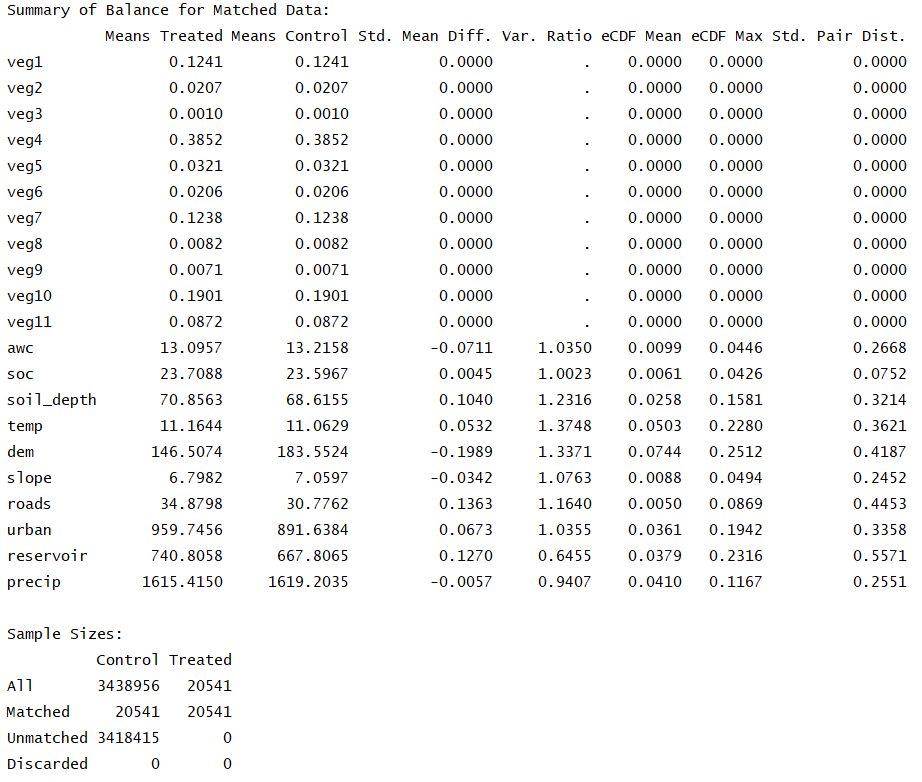
**

**Gain – Private reserves**

**
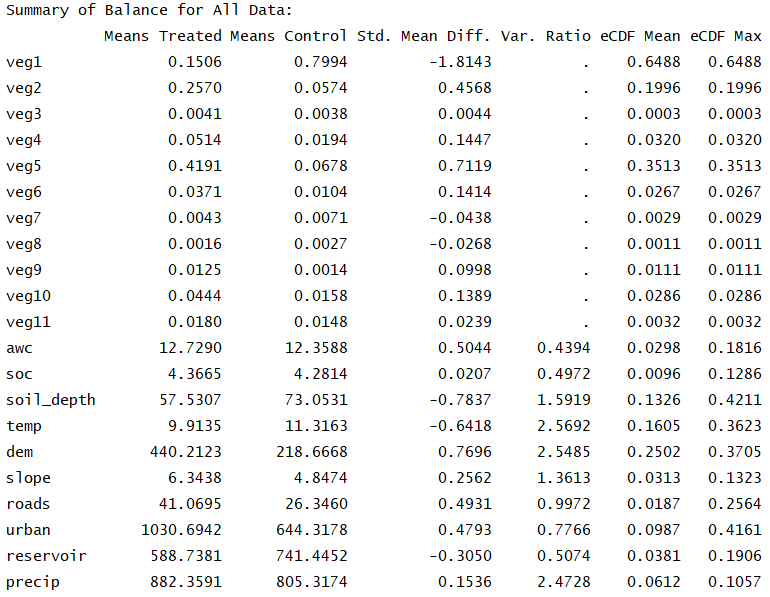
**

**
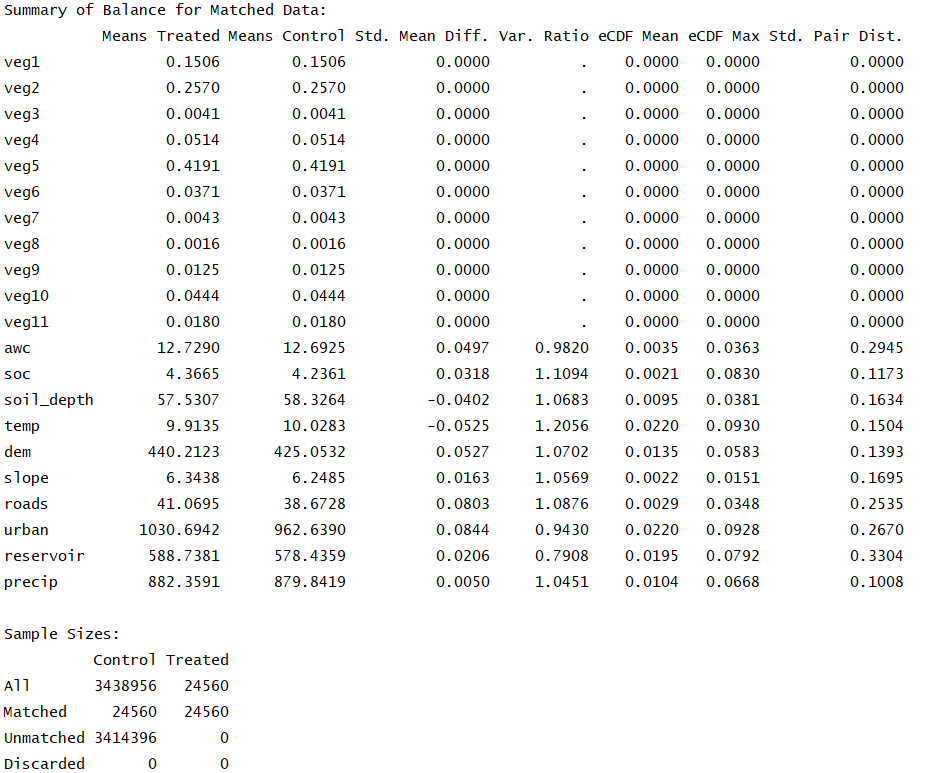
**

**Gain – Public reserves**

**
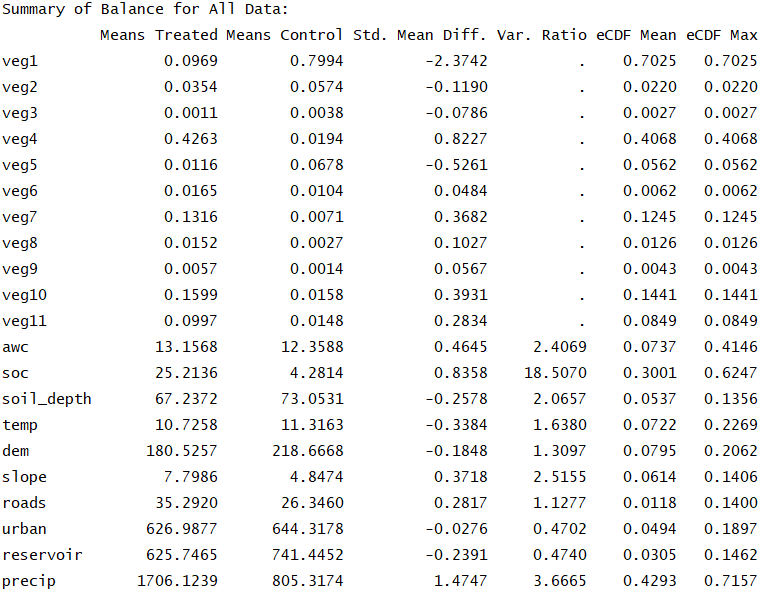
**

**
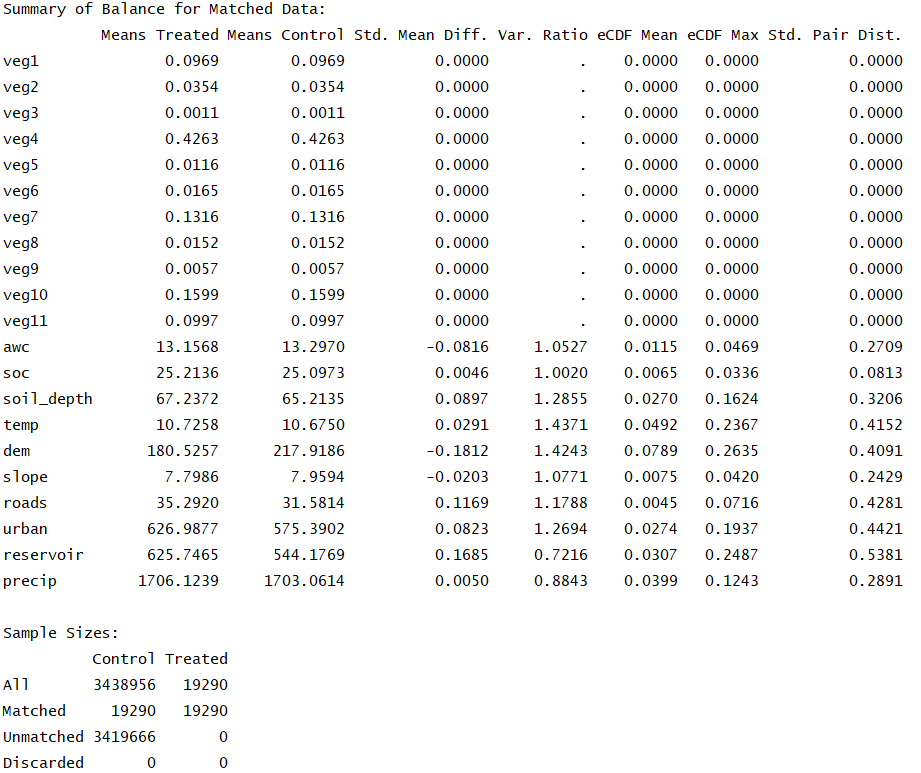
**

**Appendix S6**: Details regarding matched samples and effect estimates.
